# Resilienz gegen IT-Angriffe an Kliniken: Ergebnisse einer Stabsrahmenübung an einem Universitätsklinikum

**DOI:** 10.1007/s00101-023-01331-y

**Published:** 2023-09-19

**Authors:** E. G. Pfenninger, S. A. Schmidt, C. Rohland, S. Peters, D. McNutt, U. X. Kaisers, M. Königsdorfer

**Affiliations:** 1 Stabsstelle Katastrophenschutz, Universitätsklinikum Ulm, Albert-Einstein-Allee 29, 89081 Ulm, Deutschland; 2 Klinik für Diagnostische und Interventionelle Radiologie, Universitätsklinikum Ulm, Ulm, Deutschland; 3 Klinikumsapotheke, Universitätsklinikum Ulm, Ulm, Deutschland; 4 Zentrale Einrichtung Klinische Chemie, Universitätsklinikum Ulm, Ulm, Deutschland; 5 Zentrum für Information und Kommunikation, Universitätsklinikum Ulm, Ulm, Deutschland; 6 Klinikumsvorstand, Universitätsklinikum Ulm, Ulm, Deutschland; 7 Klinik für Anästhesiologie und Intensivmedizin, Universitätsklinikum Ulm, Ulm, Deutschland

**Keywords:** Cybersicherheit, Kritische Infrastruktur, Datensicherung, Meldepflicht, Gesundheitssystem, Cybersecurity, Critical infrastructure, Data protection, Reporting obligation, Public health system

## Abstract

**Hintergrund:**

Gesundheitssysteme, und somit auch Krankenhäuser, gehören per definitionem zur Kritischen Infrastruktur eines Landes. Vermehrt sind in den vergangenen Jahren Kliniken Ziel von Hackerangriffen mit der Folge einer wochen- bis sogar monatelangen Beeinträchtigung ihrer Handlungsfähigkeit geworden. Gemäß der „Nationalen Strategie zum Schutz Kritischer Infrastrukturen (KRITIS-Strategie)“ sind Kliniken gesetzlich verpflichtet, dagegen Vorsorge zu treffen.

**Fragestellung:**

Die vorgelegte Studie beschreibt die Planung, Durchführung und Ergebnisse einer Stabsrahmenübung an einem Großklinikum, die den Zeitraum der ersten 3 Tage bei einem hackerbedingten kompletten IT-Ausfall simulierte.

**Material und Methoden:**

In einer 8‑monatigen Evaluationsphase wurden alle IT-abhängigen Prozesse im Klinikum untersucht sowie, wenn notwendig, papierbasierte Rückfalllösungen generiert und bereichsspezifische Notfallpläne fixiert. So genannte Dienstleister, wie Apotheke, Klinische Chemie, Radiologie und Rechenzentrum, beübten einen 72-stündigen IT-Ausfall; die Klinikeinsatzleitung (KEL) steuerte im selben Zeitraum in einer Stabsübung den Ablauf. Die Teilnehmer bewerteten die Übung nach ihrem Abschluss mithilfe eines Fragebogens. Daraus sowie anhand der Vor- und Nachbereitung wurden eine Resilienzmatrix entwickelt sowie ein kurz-, mittel- und langfristiger Handlungsbedarf definiert.

**Ergebnisse:**

Die Teilnehmer bewerteten die Übung mit 85 % als sinnvoll, hatten in 97 % der Fälle zur Durchführung eine ausreichende Unterstützung und in 75 % der Fälle genügend Informationen erhalten. Dagegen fühlten sie sich persönlich und die Klinik insgesamt nur in 34 % der Fälle genügend auf einen IT-Komplettausfall vorbereitet. Die IT-ausfallsbezogenen bereichsspezifischen Notfallpläne waren vor der Übung am Klinikum in 1,7 % der Einheiten vorhanden, zur und nach der Übung in 86,7 %. Die höchste Resilienz gegenüber einem IT-Komplettausfall zeigten Einheiten, die noch auf Papierbasis arbeiteten, die geringste naturgemäß das Rechenzentrum mit komplettem Stillstand.

**Schlussfolgerung:**

Die Evaluationsphase mit der Generierung von entsprechenden Rückfallebenen ist die wichtigste Komponente in der Stärkung der Resilienz gegenüber einem Hackerangriff auf die Klinik-IT. Diese sogfältige Vorbereitung vermag die fatalen Auswirkungen auf Patienten, Personal und die gesamte Klinik zu minimieren.

## Abstract (en)

**Background:**

According to the legal definition healthcare systems and their components (e.g., hospitals) are part of the critical infrastructure of modern industrial nations. During the last few years hospitals increasingly became targets of cyber attacks causing severe impairment of their operability for weeks or even months. According to the German federal strategy for protection of critical infrastructures (KRITIS strategy), hospitals are obligated to take precautions against potential cyber attacks or other IT incidents.

**Objective:**

This article describes the process of planning, execution and results of an advanced table-top exercise which took place in a university hospital in Germany and simulated the first 3 days after a cyber attack causing a total failure of highly critical IT systems.

**Material and methods:**

During a first stage lasting about 8 months IT-dependent processes within the clinical routine were identified and analyzed. Then paper-based and off-line back-up processes and workarounds were developed and department-specific emergency plans were defined. Finally, selected central facilities such as pharmacy, laboratory, radiology, IT and the hospitals crisis management team took part in the actual disaster exercise. Afterwards the participants were asked to evaluate the exercise and the hospitals cyber security using a questionnaire. On this basis the authors visualized the hospitalʼs resilience against cyber incidents and defined short-term, medium-term and long-term needs for action.

**Results:**

Of the participants 85% assessed the exercise as beneficial, 97% indicated that they received adequate support during the preparations and 75% had received sufficient information; however, only 34% had the opinion that the hospitalʼs and their own preparedness against critical IT failures were sufficient. Before the exercise took place, IT-specific emergency plans were present only in 1.7% of the hospital facilities but after the exercise in 86.7% of the clinical and technical departments. The highest resilience against cyber attacks was not surprisingly reported by facilities that still work routinely with paper-based or off-line processes, the IT department showed the lowest resilience as it would come to a complete shutdown in cases of a total IT failure.

**Conclusion:**

The authors concluded that the planning phase is the most important stage of developing the whole exercise, giving the best opportunity for working out fallback levels and workarounds and through this strengthen the hospitals resilience against cyber attacks and comparable incidents. A meticulous preparedness can minimize the severe effects a total IT failure can cause on patient care, staff and the hospital as a whole.

## Einleitung

Das Gesundheitswesen, und somit auch Krankenhäuser, zählt zur Kritischen Infrastruktur eines Landes [1]. Kritische Infrastrukturen sind Organisationen oder Einrichtungen mit wichtiger Bedeutung für das staatliche Gemeinwesen, bei deren Ausfall oder Beeinträchtigung nachhaltig wirkende Versorgungsengpässe, erhebliche Störungen der öffentlichen Sicherheit oder andere dramatische Folgen eintreten würden [2]. Das „Zweite Gesetz zur Erhöhung der Sicherheit informationstechnischer Systeme“ (2. ITSiG, kurz auch IT-Sicherheitsgesetz 2.0) benennt neben dem Gesundheitssektor die Informationstechnik (IT) und Telekomunikation (TK) als Kritische Infrastrukturen [3]. Gerade die IT ist zunehmend Angriffen mit Schadsoftware ausgesetzt. Allein 2021 sind über 20.000 Schwachstellen in Softwareprodukten bekannt geworden (darunter 13 % als kritisch bewertet; [4]). Angriffe auf IT-Strukturen werden mit verschiedensten Methoden durchgeführt; hervorzuheben ist der Angriff mit sog. Ransomware [5]. Gemäß der Deutschen Krankenhausgesellschaft stellen Ransomware-Angriffe die größte Bedrohung für die Krankenhaus-IT dar. Eingeschleuste Ransomware verschlüsselt gespeicherte Daten und entwendet diese evtl. mit der Drohung, sie zu veröffentlichen. Ziel der Attacke ist meist eine Lösegeldforderung [5].

„Betreiber Kritischer Infrastrukturen sind verpflichtet, … angemessene organisatorische und technische Vorkehrungen zur Vermeidung von Störungen der Verfügbarkeit, Integrität, Authentizität und Vertraulichkeit ihrer informationstechnischen Systeme, Komponenten oder Prozesse zu treffen“ [3]. Zur Kritischen Infrastruktur gehören gemäß § 6 der Verordnung zur Bestimmung Kritischer Infrastrukturen nach dem BSI-Gesetz (BSI-KritisV; BSI: Bundesamt für Sicherheit in der Informationstechnik) Krankenhäuser mit mehr als 30.000 vollstationären Behandlungsfällen pro Jahr. Jedoch sind durch § 75c SGB V seit Anfang 2022 auch kleinere Häuser zur Absicherung ihrer IT-Systeme nach dem Stand der Technik verpflichtet worden [6]. Darüber hinaus kam ab 01.05.2023 hinzu, dass nach § 8a Abs. 1a des BSI-Gesetzes technische Vorkehrungen zum Einsatz von Systemen zur Angriffserkennung erfasst werden müssen. Die geeigneten Parameter und Merkmale aus dem laufenden Betrieb müssen kontinuierlich und automatisch erfasst und ausgewertet werden [7].

Nach einem erfolgreichen IT-Angriff auf die Klinikserver ist ein Krankenhaus nur noch eingeschränkt handlungsfähig, da heutzutage vielfältige Prozessabläufe EDV-gesteuert erfolgen. Cyberangriffe auf Klinken, wie sie schon mehrfach in Deutschland (z. B. in Neuss, Arnsberg, Düsseldorf, Friedrichshafen, Sigmaringen, Braunschweig und Dresden) stattgefunden haben, legen Kliniken über Wochen lahm und haben sogar schon zu einem Todesfall geführt [8, 9]. Gemäß der „Nationalen Strategie zum Schutz Kritischer Infrastrukturen (KRITIS-Strategie)“ sind Kliniken gesetzlich verpflichtet, dagegen Vorsorge zu treffen [1].

Ziel dieser Vorsorge in einer Klinik sollte sein, in einer detaillierten Planung Maßnahmen zu treffen, sodass in den ersten Tagen des IT-Ausfalls Strukturen eines „Notbetriebs“ etabliert werden. Diese sollen gewährleisten, dass ein dann über Wochen andauernder IT-Ausfall ohne Schädigung von Patienten oder Personal kompensiert werden kann. Die vorliegende Publikation beschreibt die Planung, Durchführung und Ergebnisse einer Stabsrahmenübung an einem Großklinikum, die den Zeitraum der ersten 72 h bei einem kompletten IT-Ausfall simulierte.

## Material und Methodik

In einem Vorlauf von 8 Monaten wurde in allen Kliniken, Abteilungen und Einrichtungen eruiert, wo IT-abhängige Prozesse implementiert sind und wo deren Ausfall zu kritischen Störungen führen würde. Daraufhin wurden in den betroffenen Bereichen Lösungen, die einen längerfristigen, IT-unabhängigen Weiterbetrieb gewährleisten können, diskutiert und entwickelt. Betroffen waren v. a. die „Dienstleister“, wie Radiologie, Klinische Chemie, Apotheke, Blutbank, Virologie, Bakteriologie, Zentrale Notaufnahme, OP/Intensivstationen und Sterilisation sowie maschinelle und personalabhängige Transportsysteme. Hier wurden papierbasierte Ersatzlösungen für Anforderungen aus einem in dieser Situation reduzierten Leistungsangebot sowie deren Befundung und Ausgabe entwickelt (Tab. 1). Allen Kliniken, Abteilungen und Einrichtungen wurde empfohlen, diese Unterlagen in ausgedruckter Form vorzuhalten. Außerdem wurden, soweit nicht vorhanden, speziell auf den jeweiligen Bereich abgestimmte Notfallkonzepte für einen EDV-Ausfall entwickelt; diese müssen ebenso in Druckform vor Ort vorliegen.

**Table 1:** 

Radiologie – Vollausfall der IT	Modalität möglich?	Darstellung	PACS	Anmeldung
Konventionelles Röntgen	Ja	An der Modalität	Nein	Anmeldeformulare
CT	Ja – Kurzbefund	An der Modalität, CD	Nein	Anmeldeformulare
MRT	Ja – Kurzbefund	An der Modalität, CD	Nein	Anmeldeformulare
Angio/Durchleuchtung	Ja – Kurzbefund	An der Modalität	Nein	Anmeldeformulare
Ultraschall	Ja	An der Modalität	Nein	Anmeldeformulare
Software für Nachverarbeitung (syngo.via, ISP, MINT)	Nein	Nachverarbeitung wie z. B. Schädelperfusion (Stroke), Dual-Energy-Auswertung (Knochenmarködem o. Ä.) Stenose-Quantifizierung, Lung-CAD, Rib unfold, Curved-MRP, 3D-VRT, RECIST o. Ä., etc.		

*CT* Computertomographie, *MRT* Magnetresonanztomographie, *PACS* Picture Archiving and Communication System, *CD* Computer Disc, *CAD* Computer Aided Design, *MRP* Material Requirement Planning, *VRT* Volume Rendering Technique, *RECIST* Response Evaluation Criteria In Solid Tumors, *syngo.via*: Siemens Healthineers GmbH, Erlangen, Deutschland; *ISP*: IntelliSpace Portal, Philips Healthcare, Hamburg, Deutschland; *MINT*: mint Lesion, MINT Medical GmbH, Heidelberg, Deutschland.

Im Klinikumsvorstand wurde beschlossen, dass der IT-Ausfall im Rechenzentrum, in der Radiologischen Klinik, in der Klinikumsapotheke und in der Zentralen Einrichtung Klinische Chemie real beübt werden solle; die Klinikeinsatzleitung (KEL) solle zugleich eine Stabsübung durchführen. Außerdem werden der KEL aus den verschiedensten Bereichen, wie Zentraler Notaufnahme, Intensivstationen, Sterilisationsabteilung usw., Meldungen eingespielt, die zu sichten, bewerten und abzuarbeiten sind. Die Übung wurde für 72 virtuelle Stunden angesetzt, die auf 3 h reale Übungszeit komprimiert werden sollten.

**Figure 1:**
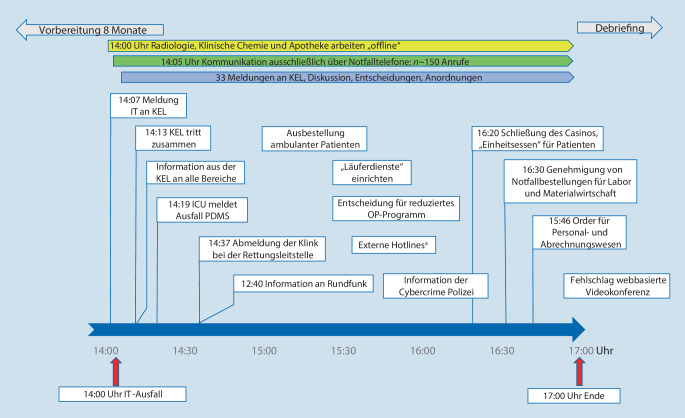

Anhand dieser Vorgaben wurde für jeden übenden Bereich ein Ablaufplan („storyboard“) entwickelt, das sowohl in realer als auch virtueller Zeit alle abzuarbeitenden Events (zu beübende Vorgänge, Ein- und Ausgänge jeglicher Art) abbildete. In die Planung waren aus jedem Bereich eine bis 2 Personen eingebunden; die übrigen betroffenen Mitarbeiter sollten unvorbereitet mit der Übung konfrontiert werden. In jeder übenden Einheit waren Beobachter und Moderatoren vor Ort. Die Planung sah einen kompletten Ausfall der IT-Infrastruktur der Klinik vor, hervorgerufen durch einen Ransomware-Angriff, wobei auch die digitale Telekommunikation betroffen sein sollte (Abb. 1).

Im Nachgang zur Übung erfolgte eine freiwillige anonyme Befragung der Übungsteilnehmer mit 18 Fragen zur Bewertung der Übung, wobei neben demografischen Daten die Kategorien „Informationen zur Übung“, „Unterstützung“, „Sinnhaftigkeit“, „Bewertung“ und „vorbereitet sein“ abgefragt wurden. Die einzelnen Fragen waren mit Schulnoten von 1 (sehr gut) bis 6 (ungenügend) zu beantworten. Der Umfrage war vom Personalrat und dem Datenschutzbeauftragten des Klinikums zugestimmt worden. Aus der Evaluation der Bereiche im Vorlauf der Übung, der „Postexercise“-Evaluation und den ausführlichen Berichten, die jede übende Einheit zu erstellen hatte, wurde eine Resilienzmatrix erstellt, wobei die Bewertung je zu einem Drittel einging.

Die gewonnenen Daten wurden in eine Excel®-Tabelle (Microsoft Corporation, Redmond, USA) übernommen und darin Mittelwerte und Standardabweichungen sowie Minima und Maxima berechnet. Die Ergebnisse werden als absolute und relative Häufigkeit dargestellt. Die Postexercise-Evaluationsbenotung wurde in Prozentzahlen transformiert, die Note „1“ gleich 100 % und die Note „6“ gleich 0 % gesetzt. Mit dem Wilcoxon-Test für unverbundene Stichproben („Origin Pro 2017, Graphing & Analysis“, Fa. OriginLab Corporation®, Northampton, MA, USA) wurden die Daten auf statistisch gesicherte Unterschiede geprüft; Korrelationen zwischen einzelnen Parametern wurden nach Spearman berechnet. Ein *p*-Wert kleiner 0,05 wurde als signifikanter Unterschied angesehen.

## Ergebnisse

In der 8‑monatigen Vorbereitungsphase ergab sich, dass zu diesem Zeitpunkt nur eine der real übenden Einheiten ein vollständiges Notfallkonzept für einen IT-Ausfall hatte, die anderen 3 hatten keine schriftliche Fixierung. Auf allen 43 bettenführenden Stationen des Klinikums gab es keinen einheitlichen IT-Notfallplan, ebenso wenig auf den 5 Intensivstationen. Viele zentralen Einrichtungen (Zentrale Interdisziplinäre Notaufnahme [ZINA], Blutbank, Virologie, Mikrobiologie) hatten ebenfalls keine schriftliche Fixierung. In den meisten Bereichen wurden deshalb spezifische Notfallpläne konzipiert (Tab. 2). In einer Intensivstation wird ein Patienten-Daten-Managementsystem (PDMS) betrieben, in 2 weiteren ist die Einführung geplant. Für das vorhandene PDMS wurde festgelegt, dass der tägliche Verordnungsplan auszudrucken sei, für die geplanten wurden die Orderspezifikation entsprechend geändert.

**Table 2:** 

Bereiche	Evaluationsphase (%)	Postexercise (%)
Intensivstationen/PACU	0/6 (0)	5/6 (83)
OP-Einheiten	0/3 (0)	2/3 (67)
Bettenstationen	0/37 (0)	37/37 (100)*
Funktionseinheiten	0/6 (0)	3/6 (50)
„Dienstleister“	1/8 (14)	7/8(86)
–	1,7	86,7*

*PACU* Post Anaesthesia Care Unit

*p < 0,05. Die Zahlen vor dem Schrägstrich geben die vorhandenen Notfallpläne an, die Zahlen hinter dem Schrägstrich sind die Anzahl der evaluierten Einheiten. in der letzten Zeile ist der prozentuale Durchschnitt angegeben

Da Erfahrungen anderer Kliniken ergaben, dass durch Ransomware bisher fast ausschließlich Windows-basierte Software-Produkte angegriffen worden waren [22], wurden Informationen von Herstellern eingeholt, inwieweit deren Geräte Windows-unabhängig sind. Großgeräte, wie digitale Röntgengeräte, CT, MRT, Laborstraßen, Konfigurations- und Fertigungsstraßen der Apotheke sowie die Warentransportanlage arbeiten im Universitätsklinikum Ulm nicht auf Windows-Basis bzw. in einem separaten, extrem abgeschirmten Umfeld und können vermutlich bedingt weiterbetrieben werden. Allerdings sind Anforderungen, Ausgabe und v. a. die Weiterverarbeitung von Bildern stark eingeschränkt bis unmöglich. Hierfür wurden netzwerkunabhängige „Stand-alone“-Rechner sowie externe Speichermedien für den notwendigen Datentransfer angeschafft. Die digitale Telekommunikationsanlage ebenso wie der Alarmserver funktionieren nicht, wohingegen die rund 170 Notfalltelefone des Klinikums funktionsfähig sind, da sie von einem externen Anbieter betrieben werden. Die Gebäudeleittechnik besitzt ein eigenes Steuerungssystem, funktioniert somit auch bei einem IT-Ausfall weiterhin. Allerdings ist geplant, dass diese in Zukunft ebenfalls über virtuelle Server im IT-Netzwerk gesteuert werden soll, und wäre dann ebenfalls betroffen.

Um den händischen Transport von anfallenden papierbasierten Anforderungs- und Befunddokumenten bewältigen zu können, wurde ein zeitlich und örtlich definierter „Rundlauf“ von Personal definiert, der alle ein- und ausgehenden Dokumente transportiert. Die Schüler der klinikeigenen Pflegeschule wurden zur Verstärkung von Schreib- und Unterstützungsarbeiten eingeplant.

**Figure 2:**
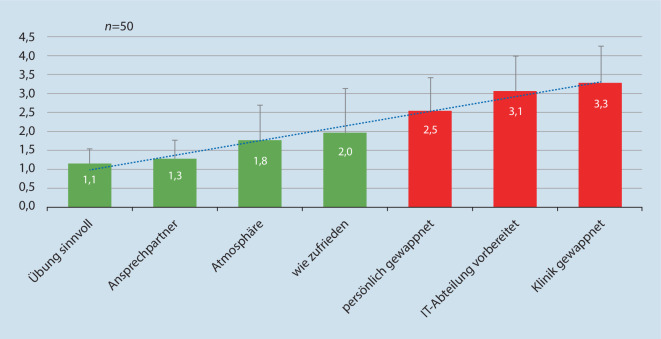

**Figure 3:**
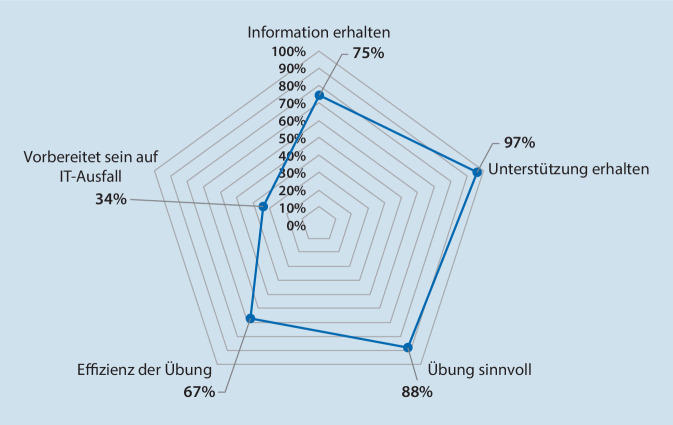

Der simulierte IT-Ausfall fand am 17.05.2022 ab 14:00 Uhr statt; die Übung wurde um 17:00 Uhr beendet. Im Anschluss erfolgte das Debriefing. Simuliert wurde ein 72-stündiger IT-Ausfall, der auf 3 h Übungszeit komprimiert wurde (Abb. 1). Repräsentative Ergebnisse der Postexercise-Befragung sind in Abb. 2 dargestellt. Die Übung wurde vorwiegend positiv bis sehr positiv gewertet, wohingegen die persönliche oder allgemeine Vorbereitung der Klinik auf einen IT-Ausfall eher kritisch beurteilt wurde. Bei der Zusammenfassung mehrerer Fragen (Clusterbewertung, Abb. 3) wurde die Sinnhaftigkeit der Übung mit 85 % positiv bewertet, die bestehenden Vorbereitungen auf einen IT-Ausfall hingegen nur mit 34 %.

**Figure 4:**
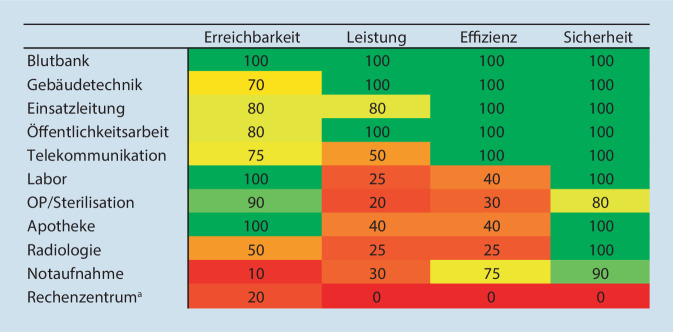

**Figure 5:**
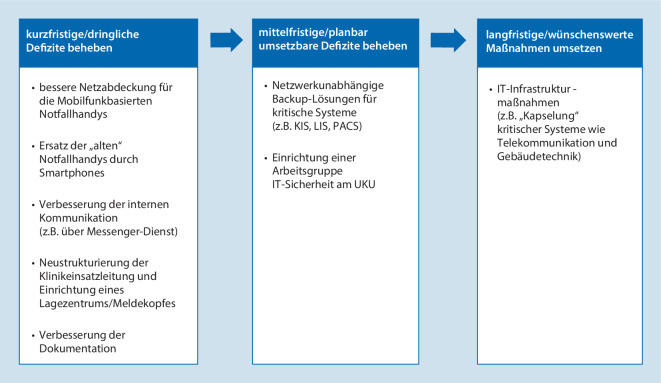

Die aus der Evaluation in der Vorbereitungsphase, der Postexercise-Befragung und den ausführlichen schriftlichen Berichten der Übenden gebildete Resilienzmatrix (Abb. 4) zeigt, dass die Blutbank, die auch jetzt noch vorwiegend auf Papierbasis arbeitet, am besten abschneidet, wohingegen das Rechenzentrum die niedrigste Resilienz besitzt. Durchschnittlich muss mit einem Rückgang der Leistungsfähigkeit der einzelnen Abteilungen auf ein Drittel bis ein Viertel des Routinebetriebs gerechnet werden. In Abb. 5 sind die kurz- und langfristig zu ergreifenden Maßnahmen aufgelistet, die sich aus der Übung ergeben haben.

## Diskussion

In der vorliegenden Studie wird anhand einer Stabsrahmenübung, die einen Ransomware-Angriff simulierte, versucht, Schwachstellen in der Resilienz eines Universitätsklinikums gegenüber einem IT-Angriff zu eruieren und daraus Folgerungen zur weiteren Bewältigung eines solchen Geschehens zu ziehen. Soweit den Autoren bekannt ist, war dies die erste IT-Ausfallsübung an einem deutschen Großklinikum und fand auch deshalb entsprechendes Interesse sowohl bei Polizei und anderen Behörden sowie bei Katastrophenschutz und Politik, die teilweise Beobachter entsandt hatten. Auch in der deutschsprachigen Literatur finden sich keine entsprechenden Publikationen. Betrachtet werden in diesem Beitrag klinische Aspekte; spezifische IT-Belange werden an anderer Stelle publiziert. Bewusst werden nicht alle Einzelheiten der durchgeführten Übung dargestellt, um potenziellen Angreifern nicht relevante Sicherheitslücken aufzuzeigen.

### Cyberangriffe auf Kliniken häufen sich

Der unmittelbarste Schaden durch die meisten Cyberangriffe weltweit betrifft immer noch Unternehmensgewinne oder die Daten von Privatpersonen, die Hacker stehlen. Die Bundesregierung hat mit dem 2. ITSiG und der BSI-KritisV eine Liste mit 9 Sektoren „Kritischer Infrastrukturen“ definiert, darunter das Gesundheitswesen, in dem ein Cyberangriff zu erheblichen Störungen und zur Schädigung der Zivilgesellschaft führen könnte [3]. Auch ohne genaue Zahlen in der Literatur zu Angaben bezüglich möglicher Todesfälle, die auf IT-Angriffe zurückzuführen sind, zeigt sich, dass Angriffe auf Krankenhäuser die Versorgung auf immer gefährlicherem Niveau stören [10]. Im Jahr 2022 wurden bei einem Angriff auf das CommonSpirit Health in den USA, dem zweitgrößten gemeinnützigen Gesundheitssystem des Landes, die persönlichen Daten von über 600.000 Patienten entwendet, einschließlich elektronischer Krankenakten. Dies führte angeblich dazu, dass einem Kind versehentlich die 5fache Dosis an Medikamenten verabreicht wurde [11]. Ein Angriff auf 3 Krankenhäuser in New York im November 2022 zwang Ärzte dazu, auf Papierlösungen umzusteigen; dies verzögerte die Patientenversorgung [12]. Erst kürzlich erfolgte ein Cyberangriff auf die „Hospital Clínic i Provincial de Barcelona“: „Wegen der Attacke habe man am Montag [06.03.2023] 150 Operationen und rund 3000 Patiententermine absagen müssen, teilten die Klinikleitung und Sprecher der katalanischen Regionalregierung …mit. Zudem seien u. a. auch die Strahlenbehandlungen von Krebskranken alle ausgefallen“ [13]. Nach Angaben des CyberPeace Institute, USA, führt ein durchschnittlicher Cyberangriff auf ein Gesundheitssystem dazu, dass Patienten 19 Tage lang keine adäquate Form der Versorgung erhalten. In einem Fall führte ein Cyberangriff zu einer rund 4‑monatigen Unterbrechung der medizinischen Versorgung [14]. Von 2016 bis 2021 hatte sich die Zahl der Ransomware-Angriffe auf das amerikanische Gesundheitssystem mehr als verdoppelt [15].

In britischen National-Health-Service-Krankenhäusern mussten nach dem globalen WannaCry-Angriff im Mai 2017 oder nach dem Angriff auf das Hollywood Presbyterian Medical Center im Februar 2016 Operationen verschoben und Patienten in nahe gelegene Krankenhäuser umgeleitet werden [16]. In Deutschland erfolgte der erste in der weiteren Öffentlichkeit bekannte Cyberangriff auf ein Großklinikum im Jahr 2020. Das Universitätsklinikum Düsseldorf war mit Ransomware angegriffen worden und musste sich für mehrere Wochen von der Notfallversorgung abmelden [8]. Zudem verstarb eine Patientin, die nicht aufgenommen werden konnte und deshalb auf erheblichem Umweg in eine andere Klinik verbracht werden musste [9]. Der russische Einmarsch in die Ukraine im Februar 2022 weckte die Sorge, dass Russland möglicherweise verheerende Cyberangriffe gegen die Ukraine, die auf benachbarte NATO-Staaten übergreifen könnten, starten würde. Ende Januar 2023 warnte das Lagezentrum der Cybersicherheitsagentur Baden-Württemberg in einer Eilmeldung die Kliniken vor möglichen russischen Angriffen auf medizinische Einrichtungen [17].

### Risikobewertungen als Indikator für Schwachstellen

In der Literatur wird ein erfolgreiches Risikomanagement durch die Schritte „hazard identification“, „risk assessment“ und „risk mitigation in case of unacceptable risk level“ definiert [18]. Für einen IT-Ausfall bedeutet dies, dass die verschiedenen Gefährdungen von Patienten, Personal und Infrastruktur, die durch den IT-Ausfall verursacht werden, identifiziert und durch Weiterentwicklung der Planung minimiert werden. Die neu gefasste ISO-31000-Norm verlagert bei der Definition von Risiko den Schwerpunkt von der Möglichkeit eines potentiellen Ereignisses, auf die Möglichkeit einer Auswirkung und insbesondere einer Auswirkung auf bestimmte Ziele [19]. In Analogie dieser ISO-Definition wurde in der 8‑monatigen Planungsphase eruiert, welche Kliniken, Abteilungen und Bereiche IT-abhängige Strukturen und Prozesse, die bei einem Cybercrimeangriff ausfallen würden, nutzen. Hierbei zeigte sich, dass in den wenigsten Bereichen der Klinik spezifische, auf den IT-Ausfall zugeschnittene Notfallkonzepte vorlagen. Zwar finden sich im Krankenhausalarm und -einsatzplan (KAEP) allgemeine Vorgehensweisen, jedoch hat sich sehr schnell gezeigt, dass spezifische Lösungen in den meisten Bereichen notwendig sind, die durch den KAEP nicht abgedeckt werden können. Das Vorgehen auf einer Intensivstation mit ihren besonderen Anforderungen und Therapieplänen unterscheidet sich grundlegend von den Bedürfnissen einer Bettenstation, in der z. B. differenzierte Diätpläne notwendig sind. Hier gehen beim IT-Ausfall in der Klinikküche keine auf die Patientenbedürfnisse abgestimmten Online-Essensbestellungen mehr ein, sodass die Essensausgabe, aber auch die Komponentenbestellungen für die Essenszubereitung in der Küche, in einem spezifisch auf diesen Bereich adaptierten Notfallplan geregelt sein muss. Dies gilt ebenso für alle anderen Unterstützungsprozesse (Apotheke, Ver- und Entsorgung, Medizintechnik, Gebäudeleittechnik und Logistik), die nicht nur auf die logistischen Prozesse abgestimmte Notfallpläne erstellen müssen, sondern auch entsprechende Offline-Papierlösungen vorzubereiten haben [20]. Da in der mehrmonatigen Evaluierungsphase deutlich wurde, dass in den wenigsten dieser Bereichen Notfallkonzepte vorhanden waren, wurden diese sowie die entsprechenden papierbasierten Dokumente erstellt. Es ergab sich somit, dass diese Evaluierungsphase mit den erarbeiteten Lösungen die wichtigste Komponente der Resilienzsteigerung gegen einen IT-Ausfall darstellte und in ihrer Bedeutung für die IT-Sicherheit die eigentliche Übung sogar übertraf.

### Übungen decken Schwachstellen der Planung auf

Der Gesetzgeber hat Kliniken darauf hingewiesen, dass sie sich der erhöhten Gefahr von Cyberangriffen bewusst sein müssen. Zu vermuten ist allerdings, dass nicht genügend Abwehrstrategien ergriffen werden [7]. Erschwerend kommt hinzu, dass Mediziner und IT-Spezialisten selten dieselbe Sprache sprechen, sodass Bedürfnis und „Awareness“ nicht immer in Einklang zu bringen sind. Hier wäre es geboten, zur Vorbereitung auf einen IT-Angriff mit nachfolgendem Ausfall der EDV gemeinsame Übungen durchzuführen. Ziel von Übungen ist u. a., „konfliktträchtige Naht- und Schnittstellen im Vorfeld zu identifizieren sowie diese zu entschärfen“ [21]. Auf europäischer Ebene führte die Agentur für Cybersicherheit der Europäischen Union (ENISA) 2022 eine Übung durch, bei der ein Angriff auf ein Gesundheitssystem simuliert wurde, um die Angriffsbereitschaft des EU-Gesundheitssektors zu bewerten [23, 24]. Eine ähnliche Übung führte die estnische Agentur für Cybersicherheit durch [25]. Insgesamt übten 918 Teilnehmer (Planer, Akteure und Beobachter) aus 27 EU-Mitgliedstaaten, 2 Ländern der Europäischen Freihandelsassoziation (EFTA) sowie mehreren EU-Institutionen und -Agenturen. Die Teilnehmer kamen aus einer Vielzahl von Sektoren; das öffentliche Gesundheitswesen stellte die meisten Teilnehmer [24]. „Die von der ENISA organisierte europaweite Übung umfasste eine Desinformationskampagne mit manipulierten Laborergebnissen und einem Cyberangriff auf europäische Krankenhausnetzwerke. Das Szenario sah vor, dass sich der Angriff zu einer EU-weiten Cyberkrise mit der unmittelbar drohenden Offenlegung personenbezogener medizinischer Daten und einer weiteren Kampagne entwickelt, die darauf abzielt, ein medizinisches, implantierbares Gerät mit der Behauptung einer Verwundbarkeit zu diskreditieren“ [22]. Allerdings sind bis jetzt (Stand Ende Februar 2023) weder Methodik noch Ergebnisse der Übung publiziert worden. Als „take away“ wurde festgehalten: „Eine Übung wie Cyber Europe wird als Trainings- und Testgrundlage benötigt, da sie erfolgreich Lücken und Entwicklungspunkte in allen Bereichen aufzeigt, um die Cybersicherheitslage aller teilnehmenden Akteure zu verbessern … Cyber Europe 2022 bestätigte die Bedeutung einer optimalen Vorbereitung auf eine solche groß angelegte Übung …. Die Übung hat bestätigt, dass häufige Tests auf lokaler Ebene notwendig sind, um die Widerstandsfähigkeit des Gesundheitssektors gegenüber Cybersicherheitsbedrohungen kontinuierlich zu verbessern und zu stärken“ [23].

Die Stabsrahmenübung hatte zum Ziel, dass nicht nur die KEL, wie meistens üblich, ein theoretisches Szenario durchspielen sollte, sondern 4 wesentliche Dienstleister des Klinikums sollten real den kompletten Ausfall des IT-Netzes mit allen Folgen beüben, nämlich das Rechenzentrum, die Radiologie, die Klinische Chemie und die Apotheke. Im täglichen klinischen Betrieb können nicht einfach ganze Abteilungen „abgeschaltet“ werden. In den direkt übenden Bereichen wurde der normale Betrieb aufrechterhalten, aber eine zweite personelle Schicht arbeitete parallel in einem Szenario, das vom Kliniknetz getrennte Geräte simulierte und die papierbasierten Ersatzlösungen benutzte. Deren Erfahrungen und Ergebnisse wurden in einem ausführlichen Report festgehalten; dieser fand Eingang sowohl in die Risikoanalyse als auch in die Festlegung kurz- und langfristig zu ergreifender Maßnahmen. Im Vorfeld der Übung hatte sich schon gezeigt, dass ein Betrieb bestimmter Geräte ohne Netz nur möglich ist, wenn zusätzliches Equipment vorgehalten wird. Zum Gerätebetrieb wurden externe Festplatten mit darauf gespeicherter Software angeschafft sowie netzwerkunabhängige Laptops mit Auswerte-Software zu Befundung und Ausgabe der Ergebnisse. Allerdings deckt das Krankenhausinformationssystem (KIS) nicht nur medizinische Prozesse wie Aufnahme und Entlassung, Diagnosen, Behandlung und Lebenserhaltungssysteme ab, sondern auch Verwaltungs‑, Finanz‑, Rechts‑, Aufzeichnungs- und alle anderen Aspekte der Informationsverarbeitung. Auch hierfür müssen entsprechende Vorhaltungen für einen längerfristigen EDV-Ausfall getroffen werden [26].

Wie schon dargelegt, geht ein IT-Ausfall mit einer potenziellen Gefährdung der Patienten einher [10]. Ein kompletter Ausfall des IT-Netzes betrifft das KIS, das Laborinformationssystem (LIS), das Radiologie-Informationssystem (RIS), das Picture Archiving and Communication System (PACS), die Telekommunikation, die OP-Planung, die Sterilisation und die Transportlogistik von Patienten und Waren [20]. Bei der Übung erfolgte deshalb frühzeitig die simulierte Abmeldung der Klinik bei der Rettungsleitstelle. Die ZINA, Ambulanzen und Radiologie arbeiten die wartenden Patienten anhand der vorgehaltenen Papiervorlagen und -dokumentation ab. Soweit möglich werden terminierte Patienten anhand von Notfallhandys und privaten mobilen Telefonen ausbestellt. Der OP-Betrieb muss in den Notfallmodus versetzt werden; dies bedeutet kein Ansetzen neuer elektiver Patienten, sondern nur noch Notfalloperationen an sich bereits in der Klinik befindenden Patienten. Letzteres stellt an und für sich kein besonderes Problem dar, da ein solches Vorgehen weitgehend dem Ablauf in der Nacht sowie an Wochenenden und Feiertagen entspricht [20]. Das Personal ist mit einer solchen Umstellung vertraut. Allerdings bringt ein IT-Ausfall noch weitergehende Einschränkungen mit sich. Alle Prozeduren müssen ausschließlich auf Papierbasis festgehalten werden, Operationen müssen ohne PACS-Einspielungen vorgenommen werden und navigatorisches Vorgehen ist weitgehend unmöglich. Ein besonderes Problem stellt der völlige Ausfall der Sterilisationsanlage dar. Ohne genügend Sterilgut ist kein sicheres Operieren mehr möglich. Im Universitätsklinikum Ulm wurde dies in der Weise gelöst, dass ein Vertrag mit einer benachbarten Klinik geschlossen wurde, sodass die Sterilgut-Aufbereitung in einer solchen Situation von der jeweils nichtbetroffenen Klinik vorgenommen wird.

### Eine Backup-Kommunikation ist unerlässlich

Schon im normalen Alltagsgeschehen und im Besonderen in Krisensituationen ist eine funktionierende Kommunikation innerhalb der Klinik und mit der Außenwelt unabdingbar. Beim IT-bedingten Ausfall der Telekommunikation müssen vordefinierte Handlungsanweisungen festgelegt werden; Ersatzlösungen müssen unverzüglich zum Einsatz kommen. Dies betrifft hardwaremäßig die Vorhaltung von ausreichenden Notfalltelefonen, die z. B. über das öffentliche Mobilfunknetz betrieben werden. Alle Mitarbeiter des Universitätsklinikum Ulm unterliegen der verpflichtenden Anweisung, diese Notfalltelefone regelmäßig auf ihren Ladezustand zu überprüfen, bei Ausfall der Kliniktelefone die Notfallhandys unverzüglich an sich zu nehmen und die Notfall-Telefonliste auch in ausgedruckter Form vorrätig zu halten. Bei der Übung zeigte sich jedoch, dass der Mobilfunkempfang in einzelnen Teilen der verzweigten Gebäudekomplexe unsicher war, sodass nachgerüstet werden musste. Die am Ende der Übung geplante Videokonferenz über eine Cloud-basierte und damit netzwerkunabhängige App kam nicht zustande, da zu viele der Notfalltelefone nicht internetfähig waren; auch hier wurden inzwischen neue internetfähige Geräte beschafft.

Bei einem IT-Ausfall ist die Klinik von außen nicht mehr erreichbar, sei es für Besucher, Presse oder externe Dienstleister. Hier empfiehlt es sich, ankommende Besucher an einem Sammelpunkt durch kompetentes Personal zu informieren und ggf. zu führen. Über den örtlichen Rundfunk oder Fernsehsender sollte die Bevölkerung informiert werden, und eine zentrale Informationsstelle soll die Presse mit gezielten, von der KEL autorisierten Informationen versorgen. Ansonsten besteht die Gefahr, dass zu viele falsche oder missverständliche Informationen von Mitarbeitenden oder Dritten über die sozialen Medien verbreitet werden. Vonseiten der KEL sollte nicht nur eine Abmeldung der Klinik bei der Rettungsleitstelle erfolgen, sondern auch umliegende Kliniken sollten hierüber und über die evtl. Notwendigkeit, Patienten zu verlegen, informiert werden [20].

Die Resilienzmatrix (Abb. 4) zeigte die Bereiche Blutbank, Gebäudeleittechnik und KEL am besten gerüstet für einen IT-Ausfall. Dies ist nicht verwunderlich, da diese Bereiche den Rückfall auf papierbasierte Anforderungen noch im Rahmen von Störungen des Routinebetriebs kennen und des Weiteren die Gebäudeleittechnik eine vom IT-Netzwerk unabhängige Steuerung besitzt. Bei einer Integration in das bestehende Kliniknetz ist zu fordern, dass diese, wie die schon bestehende digitale Telekomunikation, im IT-Netzt so „gekapselt“ werden, sodass sie von Hackerangriffen nicht tangiert werden können. Genauso muss über eine weitere Kapselung oder zu schaffende „Insellösungen“ in den Bereichen, die essenzielle Dienstleitungen für Patienten und Personal bereitstellen, nachgedacht werden. Naturgemäß erzielt die IT selbst in den ersten Tagen die schlechteste Bewertung bezüglich ihrer Resilienz, da sie für die Klinik nicht mehr zur Verfügung steht.

### Die IT-Schnittstellen stellen Angriffspunkte dar

Die Postexercise-Befragung des übenden Personals ergab eine sehr hohe Zufriedenheit mit dem Ablauf der durchgeführten Übung. Dagegen wurde das Vertrauen sowohl in die eigene Resilienz und insbesondere in die der IT-Struktur unter dem Durchschnitt bewertet. Zum Teil verständlich wird dies, da mit der zunehmenden Digitalisierung und Vernetzung im Gesundheitswesen auch die Gefahr von IT-Schwachstellen steigt; Hacker könnten diese als Einfallstor nutzen [5]. Nicht nur die papierlose Dokumentation, die Online-Speicherung von Vitalparametern in Anästhesie und Intensivmedizin, sondern auch die Steuerung aller technischen Einrichtungen ist teilweise schon in Kliniken installiert oder wird in naher Zukunft verwirklicht werden. Zudem können sich heutzutage schon Patienten und Besucher per WLAN in das Kliniknetz einwählen; hier ist darauf zu achten, dass eine konsequente Trennung und Kapselung des „Gastzugangs“ vorgenommen wird. Die Telekommunikation unter den Mitarbeitern oder nach außen wird mehr und mehr von analog auf digital umgestellt. Zudem werden zunehmend Schnittstellen nach extern geschaffen, sei es das Einlesen der elektronischen Gesundheitskarte oder Schnittstellen zu anderen Kliniken oder Praxen zur Datenübermittlung im Rahmen der Telemedizin. Für Hacker und Cyberangreifer ist es recht einfach, Schwachpunkte zu finden und Kliniksysteme anzugreifen. Auch wenn Krankenhäuser nicht direktes Ziel eines Angriffs sind, könnten sie indirekt von einem Cybervorfall betroffen sein [7]. Das IT-Sicherheitsmanagement eines Krankenhauses muss auch darauf ausgerichtet sein, dass Produkte oder externe Dienste, die das Krankenhaus im Rahmen der Behandlung nutzt, ausfallen können. Andererseits können aber auch Lieferketten Einfallstore für einen Virenangriff darstellen. Das Risiko bezüglich der Lieferketten eines Krankenhauses kann von zahlreichen potenziellen Eintrittspunkten wie Medikamenten- und Medizinproduktlieferanten ausgehen, von installierter Software oder von Fremdpersonal, die/das sich Zugang zum IT-System verschaffen kann. Um dem zumindest teilweise vorzubeugen, müssen Betreiber Kritischer Infrastruktur seit dem 01.05.2023 nach § 8a des BSI-Gesetzes angemessene organisatorische und technische Vorkehrungen treffen, um IT-Angriffe zu erkennen [7]. Außerdem regelt § 8a, Abs. 3 BSI-Gesetz die Pflicht, alle 2 Jahre ein KRITIS-Audit durchzuführen. Das Audit soll den Nachweis erbringen, dass die IT-Sicherheit dem Stand der Technik entspricht [7]. Aber auch der Faktor Mensch in der Klinik selbst stellt einen Risikofaktor dar. Nach einer amerikanischen Studie scheinen Beschäftigte im Gesundheitswesen für Cyberangriffe anfälliger zu sein als andere Personen. Die Studie ergab, dass Beschäftigte im Gesundheitswesen auf jede siebte simulierte Phishing-E-Mail klickten [28]. Deshalb sollten auch dienstliche E‑Mails grundsätzlich nicht auf private Mobilgeräte versandt werden. Schärfung der Awareness, Schulung und die Wiederholungen von Übungen sind geboten.

### Rechtspflichten bei einem Cyberangriff

„Liegt ein Cybervorfall vor, muss das Krankenhaus umgehend Maßnahmen einleiten, um eine mögliche Beeinträchtigung des Krankenhausbetriebs zu reduzieren und den reibungslosen Klinikbetrieb wiederherzustellen“ [29]. Dazu gehört auch, dass bei einem Cyberangriff im Krankenhaus verschiedene Rechtspflichten beachtet werden, die unterschiedliche Voraussetzungen und Adressaten haben [30]. Die Nichtbefolgung insbesondere der Meldepflichten nach dem BSI-Gesetz und der Datenschutz-Grundverordnung (DS-GVO) kann Strafmaßnahmen für die Klinik zur Folge haben, die den entstandenen finanziellen Schaden noch vergrößern könnten. Für die Meldung stellt das Bundesamt für Sicherheit in der Informationstechnik (BSI) ein Melde- und Informationsportal zur Verfügung [31]. Das BSI betont auch die Wichtigkeit, eine Strafanzeige zu erstatten. Die Cybercrime-Einheiten der Polizei ergreifen u. a. Maßnahmen zur Ermittlung der Angreifer und Art des Angriffs. Für diese Ermittlungen kann, nach bisherigen Erfahrungen an angegriffenen deutschen Kliniken, der Wiederaufbau der IT-Strukturen für Tage lahmgelegt sein. Im Verlauf der vorgestellten Stabsrahmenübung wurden auf Anordnung der KEL das Bundesamt für Sicherheit in der Informationstechnik (BSI), die Cybercrime Unit der Polizei und das Ministerium für Wissenschaft, Forschung und Kunst Baden-Württemberg in seiner Funktion als staatlicher Gewährträger informiert.

Forderungen nach Präventivmaßnahmen für einen IT-Angriff stehen und fallen mit der Frage nach der Finanzierung. Erst die Klärung der Finanzierungsfrage ebnet den Weg für ernsthafte Bedarfsplanungen und die Entwicklung konkret fassbarer Konzepte. Übungen sind kostenintensiv; sowohl Bund wie auch Länder stellen keine Finanzierung zur Verfügung. Damit bleibt nur ein denkbar schlechter Weg: Die Klinikbetreiber selbst müssen die aus ihrem Versorgungsauftrag ableitbaren Opportunitätskosten für die Vorsorge auf einen IT-Angriff tragen. Da dabei nahezu unvermeidbare Qualitätsdefizite auftreten, unterstreicht die Dringlichkeit einer zeitnahen Klärung der Finanzierungsfrage [27].

## Einschränkungen

In die Übung waren neben der KEL nur 4 weitere Bereiche direkt übend einbezogen. Obwohl aus anderen Bereichen Meldungen in die KEL eingespielt wurden, ist nicht auszuschließen, dass in diesen Bereichen die Resilienz gegenüber einem potenziellen IT-Ausfall nicht genügend gestärkt wurde. Eventuell ist dort auch die Awareness für ein solches Geschehen nicht genügend im Bewusstsein verankert worden. Das IT-Netz einer Klinik kann in einer Übung nicht abgeschaltet werden, da ansonsten Leben und Gesundheit der Patienten gefährdet wären. Damit lassen sich versteckte Mängel in der Planung nicht gänzlich ausschließen. Auch wurden administrative Prozesse, seien es Personalverwaltung, Controlling oder Dienstplanung, nicht beübt, da hierzu erst Lösungen erarbeitet werden müssen. Hackerangriffe auf die Klinik-IT lassen sich nicht mit absoluter Sicherheit abwenden, da zu viele Schnittstellen in und außerhalb der Klinik vorhanden sind und kriminelle Hacker mit immer neuen Methoden versuchen, sich Zugang zu verschaffen [26].

## Fazit für die Praxis

- Zur Ertüchtigung der Resilienz gegen IT-Ausfälle in einer Klinik sollte an erster Stelle die Evaluation IT-abhängiger Prozesse stehen. Betroffen können v. a. das Krankenhaus- (KIS), Radiologie- (RIS), Laborinformationssystem (LIS), Picture Archiving and Communication System (PACS) und die Telekommunikation sein.
- In allen IT-abhängigen Bereichen sollten Notfallkonzepte für einen EDV-Ausfall ausgearbeitet werden sowie papierbasierte Ersatzlösungen („workarounds“) für benötigte Prozesse bereitstehen. Diese sollten ausreichend in ausgedruckter Form vorliegen. Zusätzlich muss der IT-Ausfall in den Krankenhausalarm- und -einsatzplan (KAEP) integriert werden.
- Zu Steigerung und Überprüfung der Resilienz sind entsprechende Übungen geeignet. Hierdurch, verbunden mit einer Rückkopplung, können Mängel und Fehler in der Planung erkannt werden.
- Hackerangriffe auf die Klinik-IT lassen sich nicht mit absoluter Sicherheit abwenden, da zu viele Schnittstellen vorhanden sind und kriminelle Hacker mit immer neuen Methoden versuchen, sich Zugang zu verschaffen. Eine sorgfältige Vorbereitung vermag jedoch die Auswirkungen auf Patienten, Personal und die gesamte Klinik zu minimieren.
